# Harnessing magnetic fields: temporal–spatial enabling in water-splitting electrocatalysis

**DOI:** 10.1039/d5sc04314j

**Published:** 2025-09-09

**Authors:** Jin-Hua Liu, Jie Zheng, Lingyun Li, Wenhua Yang, Shuaijie Wang, Yu-Ze Sun, Jun Zhang, Seeram Ramakrishna, Yun-Ze Long, Yusuke Yamauchi

**Affiliations:** a Collaborative Innovation Center for Nanomaterials & Devices, College of Physics, Qingdao University Qingdao 266071 PR China yunze.long@qdu.edu.cn; b Industrial Research Institute of Nonwovens & Technical Textiles, Shandong Center for Engineered Nonwovens (SCEN), College of Textiles Clothing, Qingdao University Qingdao 266071 PR China; c Center for Nanotechnology & Sustainability, Department of Mechanical Engineering, College of Design and Engineering, National University of Singapore 9 Engineering Drive 1 117576 Singapore; d Australian Inst. Bioengn. & Nanotechnol. AIBN, University of Queensland Brisbane Qld 4072 Australia y.yamauchi@uq.edu.au; e Department of Materials Process Engineering, Graduate School of Engineering, Nagoya University Nagoya 464-8603 Japan; f Department of Convergent Biotechnology and Advanced Materials Science, Kyung Hee University 1732 Deogyeong-daero, Giheung-gu Yongin-si Gyeonggi-do 17104 South Korea

## Abstract

While the catalytic enhancement effect of magnetic fields in electrocatalytic water splitting has been established, the underlying mechanisms and optimal application strategies remain poorly understood. Here, we present a comprehensive investigation of the effects of a magnetic field on electrocatalysis using engineered Co-Ru@RuO_2_ ferrimagnetic materials, elucidating the complex relationships among magnetic fields, spin coupling, and catalytic activity in both oxygen evolution reaction (OER) and the hydrogen evolution reaction (HER). Our systematic study reveals a threshold-dependent response: weak magnetic fields (<1 T) have a negligible impact under electrochemical steady-state conditions, whereas strong magnetic fields (>3 T) significantly alter the steady state and enhance the catalytic performance. We introduce the novel concept of temporal–spatial enabling, demonstrating that the precisely timed application of magnetic fields particularly prior to electrochemical reactions can significantly enhance catalytic efficiency in both the OER and HER. Through innovative *quasi-in situ* temperature-dependent magnetization measurements, we provide direct evidence that magnetic fields modulate the electronic spin structure of the catalyst, resulting in improved catalytic activity. These findings not only deepen our fundamental understanding of magnetic field effects in electrocatalysis but also establish a new paradigm for optimizing catalytic performance *via* strategic manipulation of magnetic fields and spin dynamics, opening promising avenues for next-generation energy conversion technologies.

## Abstract 2

While the catalytic enhancement effect of magnetic fields in electrocatalytic water splitting has been established, the underlying mechanisms and optimal application strategies remain poorly understood.

## Graphical abstract

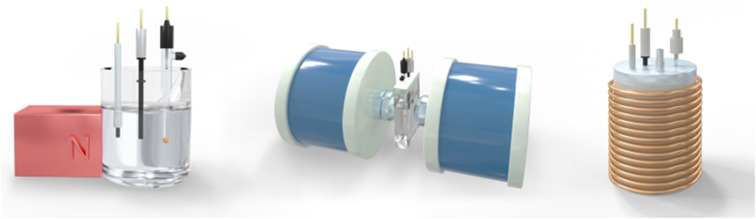

## 1. Introduction

The oxygen evolution reaction (OER) and hydrogen evolution reaction (HER) represent the fundamental half-reactions in electrochemical water splitting.^1–4^ Despite substantial progress in catalyst development—achieved through the optimization of active sites, nanostructures, and electronic properties^5–10^—the intrinsically sluggish kinetics of the OER remain a major limiting factor.^11–13^ Emerging insights into the spin-dependent characteristics of the OER have introduced an additional layer of complexity: the quantum mechanical mismatch between the singlet state of H_2_O/OH^−^ reactants and the triplet state of the O_2_ product results in a spin blockade that fundamentally constrains the reaction rate.^10,14–20^

This spin-related challenge has led to a paradigm shift in catalyst design, with particular focus on the electronic structure of 3d transition metal-based catalysts.^1,21,22^ While traditional approaches such as doping and heterojunction construction have shown promise in modulating spin states,^23–25^ they offer limited control over spin dynamics.^26,27^ External magnetic fields have emerged as a powerful alternative,^28–32^ enabling precise manipulation of the electron spin orientation and potentially overcoming spin-related limitations.^33–36^

Recent studies have demonstrated magnetic field-enhanced catalysis through various mechanisms,^9^ including magnetohydrodynamic effect,^35,37^ magnetothermal effect,^38^ and magnetic field induced spin catalysis effect.^39–41^ However, critical questions remain unresolved: What defines the optimal magnetic field strength? How does the timing of magnetic field application influence its catalytic efficacy? What are the quantitative boundaries of magnetic enhancement? These knowledge gaps are further exacerbated by the absence of direct characterisation techniques capable of probing spin phenomena during catalysis.^35,42^

In this study, we address these challenges through the development of Co-doped Ru@RuO_2_ ferrimagnetic materials and systematic investigations of magnetic field effects up to 5 T – the highest field strength reported for such studies to date. Our work introduces the novel concept of “temporal–spatial enabling”, which demonstrates that magnetic field effects are not static but depend critically on application timing. As reported by Ma *et al.*,^34^ the magnetic field is effective before CV activation. However, the electrochemical steady state can be disrupted by the application of an extremely strong magnetic field. Through innovative *quasi in situ* magnetic moment-temperature measurements, we establish a direct correlation between the magnetic moment of the catalyst and its performance, demonstrating that external magnetic fields can induce spin state rearrangement and thereby enhance catalytic activity. This comprehensive investigation deepens the fundamental understanding of magnetic field effects in electrocatalysis and establishes a robust framework for optimising catalytic processes through the strategic manipulation of spin dynamics.

## 2. Results

**Fig. 1:**
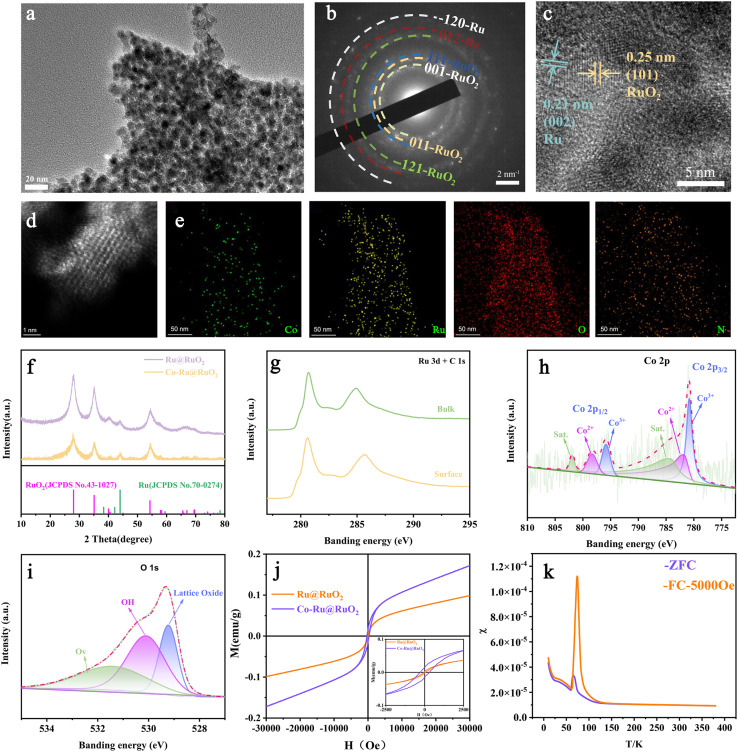
Structural characterisation of Co-Ru@RuO_2_. (a–c) HE-TEM images and SAED. (d) Aberration-corrected HAADF-STEM. (e) Elemental mapping images of Co, Ru, O, and N. (f) XRD pattern. (g–i) XPS spectra of Ru 3d + C 1s, Co 2p, and O 1s. (j) Magnetic hysteresis loops recorded at room temperature. (k) Temperature-dependent magnetisation characterisation.

The Ru-Co@RuO_2_ composite nanosheets were successfully synthesized through a multistep process involving the thermal annealing of a Ru^3+^/Co^2+^ melamine precursor in an inert atmosphere,^43^ followed by carbothermal reduction and low-temperature oxidation in air, as evidenced by the scanning transmission electron microscopy (STEM) image presented in Fig. 1a and S1a–c. The average diameter of the Co-Ru@RuO_2_ nanoparticles was 3.04 nm (Fig. S1d). The electron diffraction patterns and lattice spacings shown in Fig. 1b, c, S2a, b, and S3 confirmed the coexistence of RuO_2_, Ru and Co phases. Aberration-corrected high-angle annular dark-field scanning transmission electron microscopy (HAADF-STEM) imaging, depicted in Fig. 1d, S4 and S5, further revealed the widespread presence of a core–shell structure. The presence of carbon (Fig. S6) acts as an electron reservoir, enhancing the anticorrosion ability and stability of the Ru-based material.^44^ In addition, nitrogen derived from the melamine precursor is incorporated into the carbon matrix, increasing the conductivity.

The corresponding elemental mapping, presented in Fig. 1e, demonstrates the uniform distribution of Co, N, Ru, and O within the composite structure, indicating the successful incorporation of Co into the Ru@RuO_2_ framework. X-ray diffraction (XRD) patterns, shown in Fig. 1f confirm the coexistence of RuO_2_ (PDF#43-1027) and Ru (PDF#70-0274) phases. The similarity between the XRD patterns of Co-Ru@RuO_2_ and Ru@RuO_2_ suggests that Co anchoring was achieved without disrupting the primary Ru@RuO_2_ structure. Additionally, the diffraction peak of Ru at 44° (2*θ*), attributed to the smaller atomic radius of Co compared to Ru, indicates partial substitution of Ru by Co. To investigate the chemical states of Co-Ru@RuO_2_, X-ray photoelectron spectroscopy (XPS) was conducted. Surface etching was employed to distinguish between surface and bulk chemical states. The intensification and positive shift of the Ru 3d_5/2_ peak, as shown in Fig. 1g, indicate a relative increase in metallic Ru content at the surface. Moreover, Co was detected only in the bulk, suggesting its successful integration within the framework. The surface Co 2p spectra (Fig. S6a–d) exhibited negligible Co signals. The Co 2p spectrum presented in Fig. 1h displays characteristic Co 2p_3/2_ and Co 2p_1/2_ peaks, corresponding to a mixed oxidation state of Co^3+^ (779.0 eV and 793.8 eV) and Co^2+^ (782.1 eV and 796.0 eV). The O 1s peaks observed at 529.2 eV, 530.1 eV, and 531.4 eV in Fig. 1i are attributed to lattice oxygen, surface hydroxyl groups (OH^−^), and oxygen vacancies (Ov), respectively.

The magnetic properties of Co-Ru@RuO_2_ were characterized *via* a vibrating sample magnetometer (VSM) and a superconducting quantum interference device (SQUID). The material displayed weak residual magnetization and moderate saturation magnetization at room temperature, as shown in Fig. 1j. The introduction of Co resulted in stronger magnetic responses, which is consistent with the expected enhancement in magnetic properties. Furthermore, the *M*–*T* curve, measured under field-cooled (FC) and zero-field-cooled (ZFC) conditions and presented in Fig. 1k, demonstrated ferrimagnetic behavior for Co-Ru@RuO_2_. The magnetism under an applied field arises from a combination of the ferromagnetic/paramagnetic contribution of Co^45,46^ and the antiferromagnetic/paramagnetic behavior of Ru@RuO_2_.^45,47,48^ Notably, both antiferromagnetic and ferromagnetic materials exhibit magnetic ordering; however, in antiferromagnetic materials, the magnetic moments of neighbouring ions are aligned antiparallel and are equal in magnitude. At temperatures above the magnetic transition temperature, the Ru@RuO_2_ phase undergoes a transition from antiferromagnetic to paramagnetic, as evidenced by the room-temperature magnetisation (*M*–*H*) curve. The super-exchange interaction between magnetic ions in the antiferromagnetic phase is modulated by the applied magnetic field in conjunction with Zeeman interactions. This modulation alters the spin exchange dynamics between oxygen intermediates and the ferromagnetic catalyst, thereby reducing the spin conversion barrier and facilitating spin transitions in the oxygen intermediates.

**Fig. 2:**
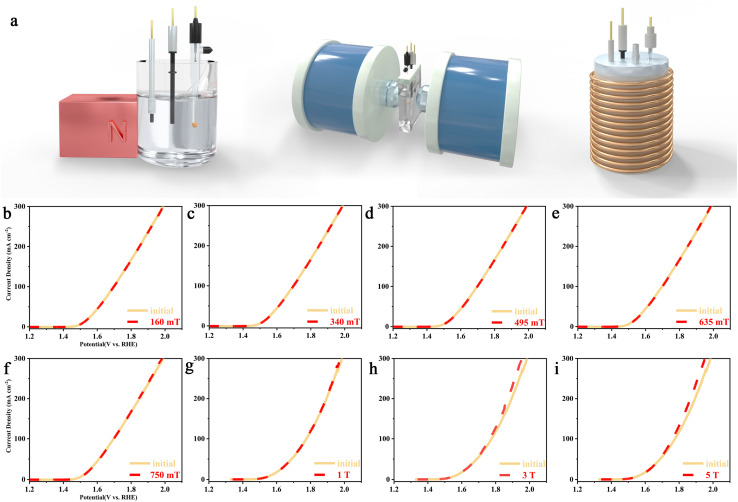
(a) Schematic illustration of the *in situ*/*operando* magnetic field assisted catalytic device (permanent magnet, DC magnetic field generator, PPMS). (b–i) Electrocatalytic OER activity in 1 M KOH under different magnetic fields.

After thoroughly confirming the formation and structural integrity of the catalysts, we conducted a comprehensive investigation into their electrocatalytic activities for the HER and OER under varying magnetic field intensities. These included weak magnetic fields generated by permanent magnets, moderate fields applied using a magnetic field generator, and strong magnetic fields provided by a Physical Property Measurement System (PPMS). The electrocatalytic assessments were executed employing a standardised three-electrode setup in a 1 M KOH solution. Both Ru@RuO_2_ and Co-Ru@RuO_2_ show good water dissociation ability (Fig. S8). Compared with Ru@RuO_2_, the Co-Ru@RuO_2_ catalyst exhibits significantly enhanced electrocatalytic performance. The incorporation of Co effectively reduces the overpotentials for both HER and OER, along with a decreased charge-transfer impedance and improved Tafel kinetics, indicating accelerated reaction rates. Furthermore, CV measurements reveal that the introduction of Co contributes to a larger electrochemically double-layer capacitance (*C*_dl_). Notably, in this section, a magnetic field was applied after the stabilization of the linear sweep voltammetry (LSV) curve, as shown in Fig. 2a. As illustrated in Fig. S9, the electrolyte temperature remained nearly constant under both permanent magnets and direct current (DC) magnetic fields. The magnetohydrodynamic (MHD) effects, driven by the Lorentz force, were observed through high-speed imaging, which revealed bubble rotation during both the HER and OER processes (Fig S10 and Video S1, SI). The Lorentz force, which continually acts perpendicularly to the motion of charged ions, facilitated their migration along circular trajectories, effectively functioning as micro-stirrers. We evaluated the OER performance of Ru@RuO_2_ under an external magnetic field, along with the HER and OER electrocatalytic stability of Co-Ru@RuO_2_ under similar conditions, as illustrated in Fig. S11. While existing literature predominantly suggests that magnetic fields exert a more pronounced regulatory effect on the OER compared to the HER—potentially due to spin exchange interactions that induce spin polarisation—our results deviated from this trend. Neither MHD effects nor spin-related effects produced a significant impact on catalytic performance, as the expected magnetic field enhancement appeared negligible (Fig. S11). We hypothesise that this discrepancy may be attributed to the specific magnetic field strength employed in these experiments.

To explore this phenomenon further, we incorporated an *in situ* electrochemical cell into the PPMS system, which was specifically designed to probe the effects of strong magnetic fields (Fig. S12). Importantly, the volume of the PPMS cavity and the *in situ* electrochemical cell required the use of Ag/AgCl as the reference electrode in a 1.0 M KOH solution. While strong alkaline conditions can influence the potential of the Ag/AgCl reference electrode, our objective was to investigate the mechanistic effects of strong magnetic fields. To ensure data accuracy, electrodes were regularly replaced. The LSV results, presented in Fig. 2b–i, revealed compelling insights. At magnetic field strengths below 1 T, no appreciable change in the OER activity of the catalyst was observed. However, when the field strength exceeded 3 T, a marked enhancement in OER activity was detected, particularly at current densities above 100 mA cm^−2^. Impedance spectroscopy and electrochemical double-layer capacitance (*C*_dl_) measurements under strong magnetic fields, shown in Fig. S13 and S14, indicate that both the solution's ohmic resistance and the *C*_dl_ remained nearly constant across varying magnetic field strengths. These findings suggest that only extremely strong magnetic fields exert a meaningful influence on the catalytic process, which may impose practical constraints on the broader application of magnetic field-assisted catalysis. This observation highlights the complexity of magnetic field interactions with electrocatalytic systems and underscores the need for further investigation to elucidate the underlying mechanisms governing these effects.

To further investigate the influence of magnetic fields on catalytic processes, a magnetic field was applied prior to the initiation of the electrochemical reaction. Specifically, the magnetic field was introduced and maintained before the commencement of the electrochemical program, and all magnetic field-related tests were performed prior to the onset of the reaction. Notably, our observations deviated significantly from those reported in Fig. 2b. Pre-reaction exposure to the magnetic field was found to modulate catalyst activation dynamics, influencing both the OER and HER. We term this newly identified phenomenon “temporal–spatial enabling.”

**Fig. 3:**
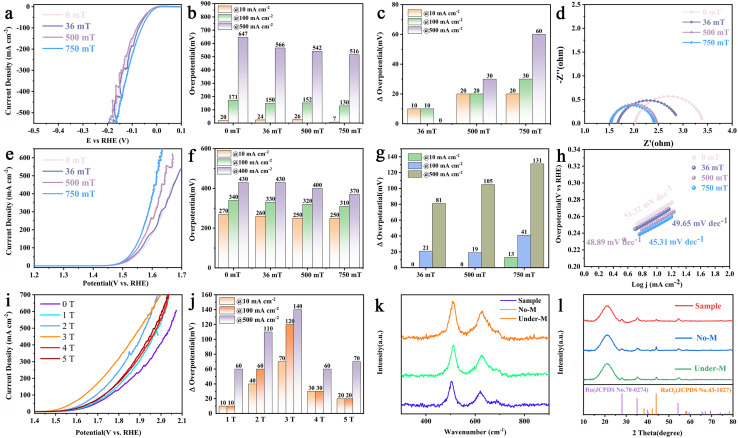
Electrochemical studies of the catalysts in 1.0 M KOH under different magnetic fields. (a) HER polarisation curves. (b) Comparison of overpotentials at 10 mA cm^−2^, 100 mA cm^−2^, and 500 mA cm^−2^. (c) HER overpotential difference caused by a magnetic field. (d) EIS slopes. (e) OER polarisation curves. (f) Comparison of overpotentials at 10 mA cm^−2^, 100 mA cm^−2^, and 400 mA cm^−2^. (g) OER overpotential difference caused by a magnetic field. (h) Tafel slopes. (i) OER polarisation curves under a strong magnetic field. (j) Comparison of overpotentials at 10 mA cm^−2^, 100 mA cm^−2^, and 500 mA cm^−2^. (k and l) Raman spectra and XRD patterns after the OER.

However, Ru@RuO_2_ exhibits minimal response under magnetic field conditions (Fig. S15a and b), thereby excluding MHD effects as a contributing factor. Independent assessments of HER and OER activities in alkaline media were subsequently conducted. LSV curves for HER, recorded in 1.0 M KOH (Fig. 3a), revealed a pronounced reduction in overpotential upon magnetic field application. Under a 750 mT magnetic field, Co-Ru@RuO_2_ demonstrated exceptionally low overpotentials of 7 mV at 10 mA cm^−2^ and 130 mV at 100 mA cm^−2^, compared to 20 mV and 171 mV, respectively, in the absence of the field (Fig. 3b). Video S2 (SI) visually confirmed the enhanced gas evolution associated with magnetic field exposure, corroborating the LSV findings. To exclude potential contributions from the counter electrode, OER activity was evaluated across a range of magnetic field intensities. Comparative analyses of catalytic performance with and without magnetic field application are summarised in Fig. 3c. The increase in catalysis correlated positively with magnetic field strength, with the effect intensifying at higher current densities. The impedance spectroscopy results (Fig. 3d) further indicated that the magnetic field facilitated the charge transfer kinetics.

Similarly, the OER LSV curves (Fig. 3e–g) in 1.0 M KOH exhibited a notable reduction in OER overpotential under a 750 mT magnetic field. Specifically, the overpotential decreased from 270 mV to 250 mV at 10 mA cm^−2^, and from 340 mV to 310 mV at 100 mA cm^−2^. These results indicate a positive correlation between the enhancement of OER activity and both magnetic field strength and current density. Video S3 (SI) further corroborates these findings by providing visual evidence that the magnetic field facilitates gas generation, rather than merely influencing the dynamics of pre-existing bubbles. Additionally, the Tafel slope decreased from 51.32 mV dec^−1^ to 45.31 mV dec^−1^ under the applied magnetic field, indicating improved reaction kinetics.

Large magnetic field OER tests were conducted *via* a custom-designed PPMS *in situ* electrochemical cell with Ag/AgCl as the reference electrode in 1.0 M KOH. The results presented in Fig. 3i and j show that, compared with moderate field strengths (<1 T), stronger magnetic fields yielded greater catalytic improvements. However, extreme fields (5 T) may induce significant Zeeman splitting, leading to more complex spin state transitions and rearrangements. Consequently, a magnetic field strength of about 3 T emerged as an optimal balance. The OER polarisation curves in Fig. 3i are different from those in Fig. 3e. The reaction under a strong magnetic field may be jointly controlled by both kinetic and non-kinetic effects due to different reference electrodes.

To gain further insight into the magnetic field effect, we measured the *C*_dl_ (Fig. S16 and S17). In the presence of a magnetic field, the *C*_dl_ value increased slightly and was positively correlated with the magnetic field strength, indicating an increase in accessible active sites. This suggests that the magnetic field influences spin states, which in turn modify the accessibility of the active site. Our findings suggest that magnetic field-induced spin polarisation may enhance active site accessibility and improve charge transfer efficiency. Table S1 summarises several recent studies reporting enhanced HER and OER performance under the influence of magnetic fields. Catalyst stability under magnetic field conditions was evaluated (Fig. S18), revealing a sustained demagnetisation effect following the removal of the magnetic field, further confirming the ferromagnetic nature of Co. Post-reaction characterisation using Raman spectroscopy and X-ray diffraction (XRD) (Fig. 3k and l) showed no significant structural alterations, indicating that the magnetic field primarily modulated the catalyst's spin configuration without compromising its structural integrity.

**Fig. 4:**
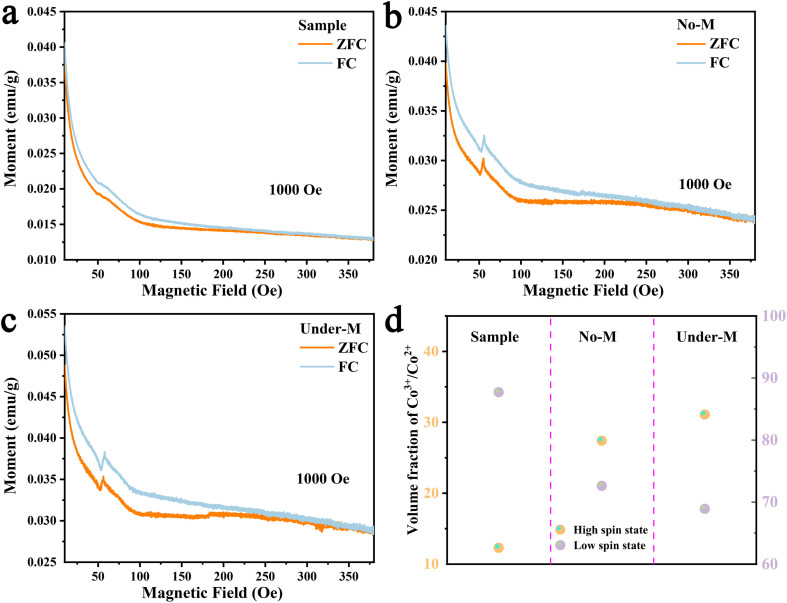
Temperature-dependent magnetisation characterisation. (a) Co-Ro@RuO_2_ catalyst (a sample). (b) The prepared catalysts after the electrochemical test (No-M). (c) The prepared catalysts in a magnetic field (750 mT) after the electrochemical test (Under-M). (d) Calculated volume fractions of HS- and LS-state Co^3+^/Co^2+^ in the four samples.

Temporal–spatial expansion affects both the HER and OER processes. We hypothesise that the concept of “temporal–spatial enabling” primarily influences the catalyst itself rather than targeting specific reaction pathways (*e.g.*, the OER). To explore the potential correlation between magnetic moments and enhanced spin polarisation within the catalyst, we performed *quasi-in situ* field-cooled *M*–*T* measurements (Fig. 4a–c) and calculated the effective magnetic moment *μ*_eff_. The catalysts were categorised as follows: the as-prepared Co-Ru@RuO_2_ (denoted as Sample), the catalyst after standard electrochemical testing (denoted as No-M), and the catalyst subjected to “temporal–spatial enabling” electrochemical testing (denoted as Under-M). Prior to *M*–*T* measurements, all catalysts were ultrasonically exfoliated and dried. Untested catalysts were also prepared into ink, drop-cast, and subjected to identical ultrasonic exfoliation and drying procedures to ensure consistency and data comparability. Although the catalyst is ferrimagnetic, its high-temperature magnetic behaviour could still be fitted using the Curie–Weiss law.
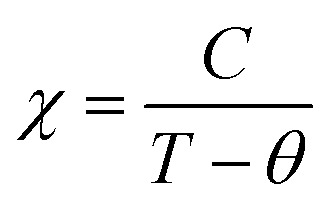

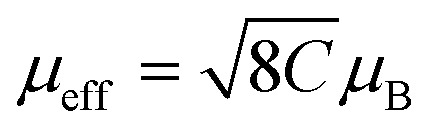
where *θ* is the Curie–Weiss temperature, *μ*_eff_ is the effective magnetic moment, *μ*_B_ is the Bohr magneton, and *C* is the Curie constant. The results (Fig. 4d) show that after electrochemical reactions in the absence of a magnetic field, the effective magnetic moment of the catalyst increases, even though its structure remains unchanged. For catalysts subjected to “temporal–spatial enabling”, the effective magnetic moment further increases post-reaction compared with that of samples without magnetic fields. This finding indicates that the intrinsic feature of “temporal–spatial enabling” is the magnetic-field-induced alteration of the effective magnetic moment. The effective magnetic moment can be used to calculate the ratio of high-spin (HS) to low-spin (LS) magnetic ions:^49^
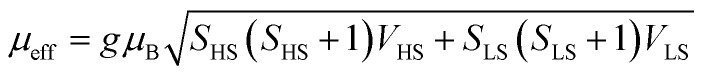
*V*_HS_ + *V*_LS_ = 1Here, the Landé factor *g* = 2, *V*_HS_ and *V*_LS_ are the volume fractions of Co^3+^ in the high-spin state (HS) and low-spin state (LS), respectively, and *S* is the total spin quantum number.^50–52^ The magnetic field interacts with the electron spin and orbital angular momentum of cobalt ions, thereby influencing electron spin states.

By analysing the bond energy relationships between Co^2+^/Co^3+^ and the reactants (Fig. S19–S22), we identified that only changes in the spin state of Co^3+^ significantly influenced the OER activity. A marked increase in the proportion of high-spin Co^3+^ states was observed, which likely contributes to the enhanced OER performance. These results suggest that the “temporal–spatial enabling” effect modifies the magnetic properties of the catalyst by promoting a transition from low-spin to high-spin states, thereby enhancing both spin polarisation and catalytic efficiency.

The results shown in Fig. 2b indicate that the “enabling” effect is temporally limited; it diminishes markedly once the catalytic system reaches a steady-state reaction condition. In our system, the ferrimagnetic catalyst attains magnetic saturation under moderate external fields, indicating that spin polarisation or spin state transfer alone does not comprehensively account for the observed impacts of strong external magnetic fields on the HER and OER processes. The results of density functional theory calculations corroborate this observation (Fig. S23–S26). Furthermore, under strong magnetic fields, the orbital motion of electrons may become quantised, substantially restricting both their mobility and accessible energy states. Under these conditions, the stability of reaction intermediates and even the reaction pathways may be altered, potentially resulting in changes to reaction products or kinetics. This phenomenon demands a thorough investigation, requiring the application of advanced characterisation techniques to fully elucidate and understand the underlying mechanisms and effects.

## 3. Conclusion

In this study, we successfully designed and evaluated a Co-Ru@RuO_2_ ferrimagnetic catalyst to explore the effects of magnetic fields on the electrochemical processes of the HER and OER. By systematically comparing the effects of magnetic fields applied before and after the onset of electrochemical reactions, we identified a significant increase in catalytic activity only when a magnetic field was introduced prior to reaction initiation. This “temporal–spatial enabling” phenomenon suggests that magnetic fields can modulate catalyst activation dynamics, leading to improved charge transfer kinetics, lower overpotentials, and enhanced gas evolution. Notably, at a magnetic field strength of 750 mT, the Co-Ru@RuO_2_ catalyst demonstrates outstanding electrocatalytic performance, exhibiting a HER overpotential of 7 mV and an OER overpotential of 250 mV at 10 mA cm^−2^. Additionally, our *in situ M*–*T* measurements reveal a compelling phenomenon: the electrochemical reaction induces an increase in the catalyst's effective magnetic moment, which is further enhanced by the applied magnetic field. This observation points to a potential mechanism for magnetic-field-assisted electrocatalysis, wherein the magnetic field interacts with the intrinsic magnetic properties of the catalyst to elevate its catalytic activity. Our findings offer novel insights into the mechanisms governing magnetic-field-assisted electrocatalysis for both HER and OER, contributing to the advancement of more efficient and sustainable energy conversion systems.

## Author contributions

J. H. L. conceived the idea and wrote the manuscript. L. L., S. W., Y. Z. S. mainly completed the experimental part. J. Z. and S. R. participated in the experiments. Y. Z. L. guided the project. W. Y. analyzed the data. J. Z., Y. Z. L. and Y. Y. revised the manuscript.

## Conflicts of interest

The authors declare that they have no known competing financial interests or personal relationships that could have appeared to influence the work reported in this paper.

## Supplementary Material

SC-016-D5SC04314J-s001

SC-016-D5SC04314J-s002

SC-016-D5SC04314J-s003

SC-016-D5SC04314J-s004

## Data Availability

All experimental data supporting the findings of this study are available within the article and its SI. See DOI: https://doi.org/10.1039/d5sc04314j.

## References

[cit1] Zhang Y., Wu Q., Seow J. Z. Y., Jia Y., Ren X., Xu Z. J. (2024). Chem. Soc. Rev..

[cit2] Wang Q., Oldham L. I., Giner-Requena A., Wang Z., Benetti D., Montilla-Verdu S., Chen R., Du D., Lana-Villarreal T., Aschauer U., Guijarro N., Durrant J. R., Luo J. (2024). J. Am. Chem. Soc..

[cit3] Yang M., Ding J., Wang Z., Zhang J., Peng Z., Liu X. (2025). Chin. Chem. Lett..

[cit4] Liu W., Zhou M., Zhang J., Liu W., Qin D., Liu Q., Hu G., Liu X. (2025). Mater. Chem. Front..

[cit5] Chen M., Ma J., Chen C., Ding J., Liu Y., He H., Liu Q., Hu G., Wu Y., Liu X. (2024). Chem. Eng. J..

[cit6] Zhao H., Zhu L., Yin J., Jin J., Du X., Tan L., Peng Y., Xi P., Yan C. H. (2024). Angew Chem. Int. Ed. Engl..

[cit7] Zhang L., Li W., Ren S., Song W., Wang C., Lu X. (2024). Adv. Energy Mater..

[cit8] He C., Yang L., Wang J., Wang T., Ju J., Lu Y., Chen W. (2024). Carbon Energy.

[cit9] Wang Y., Li S., Hou X., Cui T., Zhuang Z., Zhao Y., Wang H., Wei W., Xu M., Fu Q., Chen C., Wang D. (2024). Adv. Mater..

[cit10] Wang S., Yao S., Zhang F., Ji K., Ji Y., Li J., Fu W., Liu Y., Yang J., Liu R., Xie J., Yang Z., Yan Y. M. (2024). Angew Chem. Int. Ed. Engl..

[cit11] Gong X., Jiang Z., Zeng W., Hu C., Luo X., Lei W., Yuan C. (2022). Nano Lett..

[cit12] Wang Y., Li Q., Wang M., Ou H., Deng D., Zheng H., Bai Y., Zheng L., Chen Z. Y., Li W., Fang G., Lei Y. (2024). Nano Lett..

[cit13] Lee W. H., Han M. H., Ko Y. J., Min B. K., Chae K. H., Oh H. S. (2022). Nat. Commun..

[cit14] Zhang Z., Ma P., Luo L., Ding X., Zhou S., Zeng J. (2023). Angew Chem. Int. Ed. Engl..

[cit15] Hunt C., Zhang Z., Ocean K., Jansonius R. P., Abbas M., Dvorak D. J., Kurimoto A., Lees E. W., Ghosh S., Turkiewicz A., Garces Pineda F. A., Fork D. K., Berlinguette C. P. (2022). J. Am. Chem. Soc..

[cit16] Yu Z., Zhang D., Wang Y., Liu F., She F., Chen J., Zhang Y., Wang R., Zeng Z., Song L., Chen Y., Li H., Wei L. (2024). Adv. Mater..

[cit17] Pan L., Ai M., Huang C., Yin L., Liu X., Zhang R., Wang S., Jiang Z., Zhang X., Zou J. J., Mi W. (2020). Nat. Commun..

[cit18] Li X., Cheng Z., Wang X. (2020). Electrochem. Energy Rev..

[cit19] Gao Y., Zhang M., Zhao Q., Liu W., Zheng L., Ouyang J., Na N. (2024). Energy Environ. Sci..

[cit20] Wang Z., Huang W., Wu H., Wu Y., Shi K., Li J., Zhang W., Liu Q. (2024). Adv. Funct. Mater..

[cit21] Suntivich J., May K. J., Gasteiger H. A., Goodenough J. B., Shao-Horn Y. (2011). Science.

[cit22] Huang Y., Li S., Zhang Z., Cui P. (2024). Phys. Rev. B.

[cit23] Sun Z., Lin L., He J., Ding D., Wang T., Li J., Li M., Liu Y., Li Y., Yuan M., Huang B., Li H., Sun G. (2022). J. Am. Chem. Soc..

[cit24] Li Z., Wang Z., Xi S., Zhao X., Sun T., Li J., Yu W., Xu H., Herng T. S., Hai X., Lyu P., Zhao M., Pennycook S. J., Ding J., Xiao H., Lu J. (2021). ACS Nano.

[cit25] Wang Y., Meng P., Yang Z., Jiang M., Yang J., Li H., Zhang J., Sun B., Fu C. (2023). Angew Chem. Int. Ed. Engl..

[cit26] Saini K., Nair A. N., Yadav A., Enriquez L. G., Pollock C. J., House S. D., Yang S., Guo X., Sreenivasan S. T. (2023). Adv. Energy Mater..

[cit27] Li L., Zhou J., Wang X., Gracia J., Valvidares M., Ke J., Fang M., Shen C., Chen J. M., Chang Y. C., Pao C. W., Hsu S. Y., Lee J. F., Ruotolo A., Chin Y., Hu Z., Huang X., Shao Q. (2023). Adv. Mater..

[cit28] Ren X., Wu T., Sun Y., Li Y., Xian G., Liu X., Shen C., Gracia J., Gao H. J., Yang H., Xu Z. J. (2021). Nat. Commun..

[cit29] Wu T., Ren X., Sun Y., Sun S., Xian G., Scherer G. G., Fisher A. C., Mandler D., Ager J. W., Grimaud A., Wang J., Shen C., Yang H., Gracia J., Gao H. J., Xu Z. J. (2021). Nat. Commun..

[cit30] Zhou G., Wang P., Li H., Hu B., Sun Y., Huang R., Liu L. (2021). Nat. Commun..

[cit31] Zhang Y., Guo P., Niu S., Wu J., Wang W., Song B., Wang X., Jiang Z., Xu P. (2022). Small Methods.

[cit32] Guo P., Zhang Y., Han F., Du Y., Song B., Wang W., Wang X., Zhou Y., Xu P. (2022). J. Phys. Chem. Lett..

[cit33] Sun T., Tang Z., Zang W., Li Z., Li J., Li Z., Cao L., Dominic Rodriguez J. S., Mariano C. O. M., Xu H., Lyu P., Hai X., Lin H., Sheng X., Shi J., Zheng Y., Lu Y. R., He Q., Chen J., Novoselov K. S., Chuang C. H., Xi S., Luo X., Lu J. (2023). Nat. Nanotechnol..

[cit34] Ma S., Wang K., Rafique M., Han J., Fu Q., Jiang S., Wang X., Yao T., Xu P., Song B. (2024). Angew. Chem., Int. Ed..

[cit35] Chen J. B., Ying J., Tian Y., Xiao Y. X., Yang X. Y. (2025). Adv. Funct. Mater..

[cit36] Huang Y., Xu H.-S. (2023). Appl. Phys. Lett..

[cit37] Vensaus P., Liang Y., Ansermet J. P., Soler-Illia G., Lingenfelder M. (2024). Nat. Commun..

[cit38] Luo L., Xu L., Wang Q., Shi Q., Zhou H., Li Z., Shao M., Duan X. (2023). Adv. Energy Mater..

[cit39] Su M. X., Zhou W. D., Liu L., Chen M. Y., Jiang Z. Z., Luo X. F., Yang Y., Yu T., Lei W., Yuan C. L. (2022). Adv. Funct. Mater..

[cit40] Wang H., Wang K., Zuo Y., Wei M., Pei P., Zhang P., Chen Z., Shang N. (2022). Adv. Funct. Mater..

[cit41] Wang K., Yang Q., Zhang H., Zhang M., Jiang H., Zheng C., Li J. (2023). J. Mater. Chem. A.

[cit42] Ren X., Wu T., Gong Z., Pan L., Meng J., Yang H., Dagbjartsdottir F. B., Fisher A., Gao H. J., Xu Z. J. (2023). Nat. Commun..

[cit43] Li Y., Wang W., Cheng M., Feng Y., Han X., Qian Q., Zhu Y., Zhang G. (2023). Adv. Mater..

[cit44] Cui X., Ren P., Ma C., Zhao J., Chen R., Chen S., Rajan N. P., Li H., Yu L., Tian Z., Deng D. (2020). Adv. Mater..

[cit45] Das M., Dutta P., Giri S., Majumdar S. (2019). J. Phys.: Condens. Matter.

[cit46] Szaller D., Prodan L., Geirhos K., Felea V., Skourski Y., Gorbunov D., Förster T., Helm T., Nomura T., Miyata A., Zherlitsyn S., Wosnitza J., Tsirlin A. A., Tsurkan V., Kézsmárki I. (2025). Phys. Rev. B.

[cit47] Zhu Z. H., Strempfer J., Rao R. R., Occhialini C. A., Pelliciari J., Choi Y., Kawaguchi T., You H., Mitchell J. F., Shao-Horn Y., Comin R. (2019). Phys. Rev. Lett..

[cit48] Feng X., Bai H., Fan X., Guo M., Zhang Z., Chai G., Wang T., Xue D., Song C., Fan X. (2024). Phys. Rev. Lett..

[cit49] Sun Y., Ren X., Sun S., Liu Z., Xi S., Xu Z. J. (2021). Angew Chem. Int. Ed. Engl..

[cit50] Chen J.-B., Ying J., Tian Y., Xiao Y.-X., Yang X.-Y. (2025). Adv. Funct. Mater..

[cit51] Zhang Y., Wu Q., Seow J. Z. Y., Jia Y., Ren X., Xu Z. J. (2024). Chem. Soc. Rev..

[cit52] Zhang C. Y., Zhang C., Sun G. W., Pan J. L., Gong L., Sun G. Z., Biendicho J. J., Balcells L., Fan X. L., Morante J. R., Zhou J. Y., Cabot A. (2022). Angew. Chem., Int. Ed..

