# Supplementary material for: Harnessing magnetic fields: temporal–spatial enabling in water-splitting electrocatalysis
Source: Chem Sci. 2025 Sep 9;16(39):18309–17. doi: 10.1039/d5sc04314j (PMC12419466; doi:10.1039/d5sc04314j)
Supplement: SC-016-D5SC04314J-s001 [file SC-016-D5SC04314J-s001.pdf]

Supplementary Information (SI) for Chemical Science.
This journal is © The Royal Society of Chemistry 2025

Supplementary Information

Harnessing Magnetic Fields: Temporal-Spatial Enabling in Water-Splitting Electrocatalysis

Jin-Hua Liu [a,1], Jie Zheng [b,1], Lingyun Li [a], Wenhua Yang [a], Shuaijie Wang [a], Yu-Ze Sun [a], Jun Zhang [a], Seeram Ramakrishna [a,c], Yun-Ze Long [a,*], Yusuke Yamauchi [d,e,f,*]

[a] *Collaborative Innovation Center for Nanomaterials & Devices, College of Physics, Qingdao University, Qingdao 266071, PR China, yunze.long@qdu.edu.cn*

[b] *Industrial Research Institute of Nonwovens & Technical Textiles, Shandong Center for Engineered Nonwovens (SCEN), College of Textiles Clothing, Qingdao University, Qingdao 266071, PR China*

[c] *Center for Nanotechnology & Sustainability, Department of Mechanical Engineering, College of Design and Engineering, National University of Singapore, 9 Engineering Drive 1 117576, Singapore*

[d] *Australian Inst Bioengn & Nanotechnol AIBN, University of Queensland, Brisbane, Qld 4072, Australia, y.yamauchi@uq.edu.au*

[e] *Department of Materials Process Engineering, Graduate School of Engineering, Nagoya University, Nagoya 464-8603, Japan*

[f] *Department of Convergent Biotechnology and Advanced Materials Science, Kyung Hee University, 1732 Deogyeong-daero, Giheung-gu, Yongin-si, Gyeonggi-do 17104, South Korea*

[*] Corresponding authors.

E-mail addresses: yunze.long@qdu.edu.cn, y.yamauchi@uq.edu.au.

[1] These two authors contributed equally to this work.

Experimental sections

Preparation of Co-Ru@RuO$_2$

0.01 mmol $CoCl_2$ and 0.09 mmol $RuCl_3$ along with 1.5 mmol of melamine, were uniformly dispersed in 15 ml of ethanol and then centrifuged to separate the precursor. The precursor was then pyrolyzed at 850 °C for 2 h under Ar atmosphere. Co-Ru@RuO$_2$ is prepared was prepared by heating the pyrolyzed material at 200 °C for 3 h in the air, with a heating rate of 2 °C min^{-1}.

Materials Characterizations

The morphology was observed via the transmission electron microscopy (TEM, FEI Tecnai G2F20). Spherical aberration correction HAADF–STEM images and the corresponding mapping images were collected on a Thermo Fisher Titan Themis G2 60-300; The crystal structure and elemental composition were characterized by powder X-ray diffraction (XRD) using Kα radiation (λ = 1.5406 Å) from 10° to 80°, and by X-ray photoelectron spectroscopy (XPS, ESCALAB Xi+). The magnetic properties of the samples were measured at different magnetic fields and temperatures using a vibrating sample magnetometer (Vibrating Sample Magnetometer, Quantum Design PPMS).

Electrochemical Characterization

Electrochemical measurements were all conducted in a typical three-electrode system at 25 °C in 1.0 M KOH using a CHI760E electrochemical station. Hg/HgO and a graphite rod were used as the reference electrode (RE) and counter electrode (CE), respectively. The carbon paper was cut into 1 × 1 cm^2 and served as the working electrode (WE). Potentials were converted to the reversible hydrogen electrode (RHE) using the equation E_{RHE} =0.0591 × pH + 0.098 V. To prepare the electrocatalyst ink, 5 mg of electrocatalyst was dispersed into a mixture of 0.485 mL of deionized water and 0.485 mL of ethanol, along with 30 μL of Nafion117 solution. After another 30 min of ultrasonication, 5 μL of the electrocatalyst ink was cast on the carbon paper and dried naturally in the air. All linear sweep voltammetry (LSV) curves were obtained at a scan rate of 5 mV s^{-1} with 90% iR-compensation. Tafel plots were obtained according to the Tafel equation:

$$\eta = \mathrm{a} + b\log j \quad (1)$$

where η, b, and j represent the overpotential, Tafel slope, and current density, respectively. Electrochemical double layer capacitances (C_{dl}) were measured by analyzing the cyclic voltammetry (CV) curves at scan rates from 20 to 100 mV s^{-1}. By plotting Δi/2 ($\Delta i = i_p - i_n$, where i_p and i_n represent the positive and negative current, respectively), the Cdl can be calculated using the equation: $C_{dl} = \Delta i/2\ v$. The electrochemical active surface area (ECSA) was estimated by the equation: ECSA = C_{dl}/C_s, where the specific capacitance value (C_s) was taken as 0.04 mF cm^{-2}.

Electrocatalytic Measurements: The alkaline OER performances with/without applying a magnetic field were measured in a CHI660e electrochemical workstation using a three-electrode set-up in 1.0 m KOH electrolyte. In the PPMS in-situ electrolysis cell, Ag/AgCl and a platinum wire acted as the reference electrode and counter electrode, respectively. In a conventional electrolytic cell, Hg/HgO and a graphite rod were used as the reference electrode and count electrode, respectively. The carbon paper coated with catalyst acted as the working electrode. The sweep rate was 5 mV s^{-1} for linear sweep voltammetry (LSV) curves. The Nernst equation was used to convert potentials from measured potentials (vs reference electrode) to RHE ($E_{RHE} = E_{Ag/AgCl} + 0.197 + 0.059 \times Ph$, with iR correction applied). Specific electrochemical impedance spectroscopy (EIS) measurements were performed to measure the electrolyte resistance R (0.5–3 Ω) and charge-transfer resistance at 1.6 V versus RHE (in the range of 10 kHz to 0.1 Hz). The CV curves were measured at different scan ratesto evaluate the electrochemical double layer capacitance (C_{dl}) value.

Computational details

All density functional theory (DFT) calculations were performed using the Vienna ab initio simulation package (VASP) [1-2]. The generalized gradient approximation (GGA) with the Perdew-Burke-Ernzerhof (PBE) functional was adopted to treat the exchange and correlation energy. The electron-ion interactions were described by projector augmented wave (PAW) method [3]. To study the mechanistic chemistry of surface reactions, a heterostructure model Ru(001)/RuO_2(110) was built, as shown in

Figure S9. In the experiment, a small amount of Co was doped into Ru. In this calculation, we considered doping with 2 Co atoms and four possible doping models (AB sites, AC sites, AD sites, AE sites) were considered, as shown in Figure S9. Among them, the fourth model (AE sites) had the lowest enthalpy value. Therefore, all adsorption of O/OH/OOH in the manuscript is based on this model. To avoid interactions between adjacent surfaces, a 10 Å vacuum layer was created. During the geometry optimization,the bottom three layers of atoms were fixed, while the other layers were allowed to relax. Appropriate Monkhorst− Pack Brillouin sampling grid with a spacing $2\pi \times 0.04$ A^{-1} and an energy cut off of 400 eV were used for all cases. The convergence energy threshold for the electronic self-consistent iteration is 10^{-5} eV.

In alkaline conditions, the OER reaction is usually assumed to involve the following four steps.

$$* +2H_2O(l)\rightarrow *OH+H^{+}+H_2O\ (l)+e^{-} \quad (2)$$

$$*OH+H_2O\rightarrow *O+H_2O(l)+H^{+}+e^{-} \quad (3)$$

$$*O +H_2O\rightarrow *OOH+H^{+}+e^{-} \quad (4)$$

$$*OOH\rightarrow *+O_2(g)+H^{+} +e^{-} \quad (5)$$

Where, * represents the heterostructure model, (*l*) and (g) represent a liquid and gas phase, respectively. *OH, *O and *OOH are important intermediate in the OER activity. The absorption energies (E_{ads}) of intermediates were calculated by the following equation.

$$\Delta E_{ads}=E_{total} - E_{slab} - E_{adsorbate} \quad (6)$$

Where E_{total}, E_{slab} and $E_{adsorbate}$ are the total energy of heterostructure model Ru(001)/RuO_2(110) with the adsorbates, the Ru(001)/RuO_2(110) slab, and the total energy of pure adsorbates in the gas phase. The reaction free energy was calculated using the following equation.

$$\Delta G=\Delta E_{ads}+\Delta ZPE-T\Delta S \quad (7)$$

Where ΔZPE and ΔS are zero-point energy difference and entropy changes, respectively, and T is the room temperature of 298.15 K.

[1] J. Perdew, J. Chevary, S. Vosko, K. Jackson, M. Pederson, D. Singh, C. Fiolhais, Atoms, molecules, solids, and surfaces: Applications of the generalize d gradient

approximation for exchange and correlation, Phys. Rev. B 46 (1992) 6671,
[2] G. Kresse, J. Furthmüller, Efficiency of ab-initio total energy calculations for metals and semiconductors using a plane-wave basis set, Comp. Mater. Sci. 6 (1996) 15–50,
[3] E. Blöchl, Projector augmented-wave method, Phys. Rev. B 50 (1994) 17953

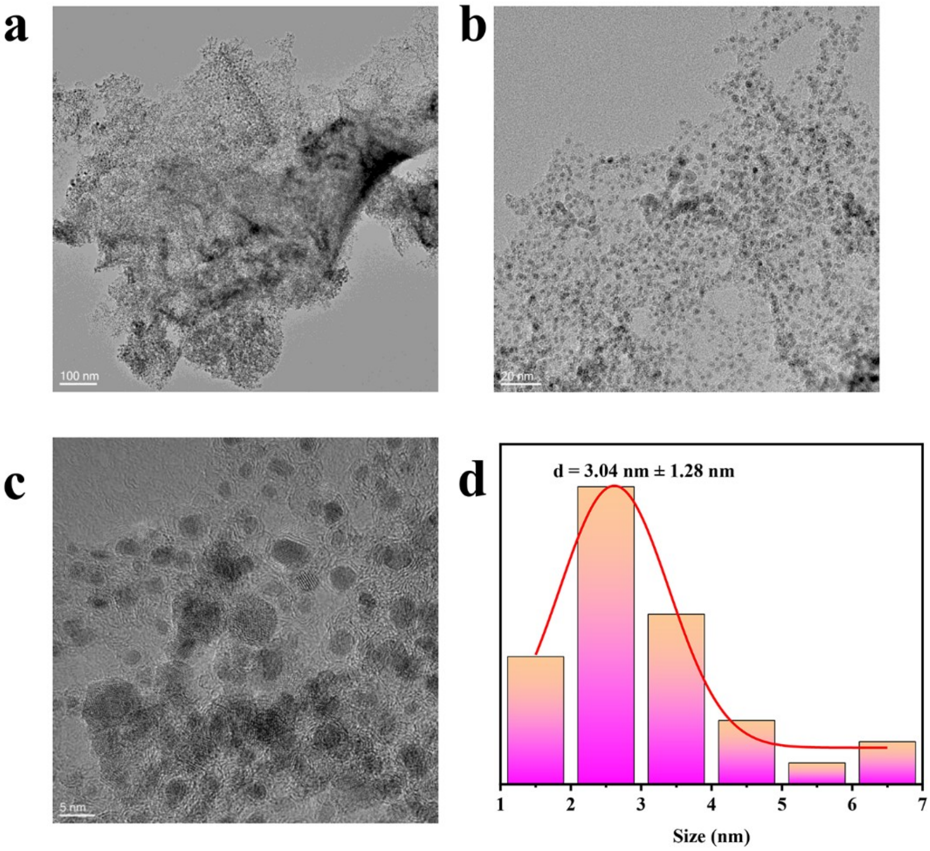

Fig. S1. a-c. High-magnification TEM images of and **d.** size statistics of Co-Ru@RuO_2.

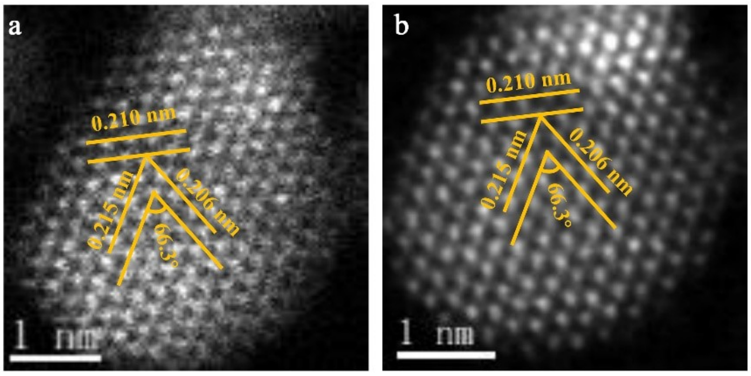

Fig. S2. a-b. The aberration corrected HAADF-STEM of two distinct locations in Co-Ru@RuO_2, showing the Ru phase.

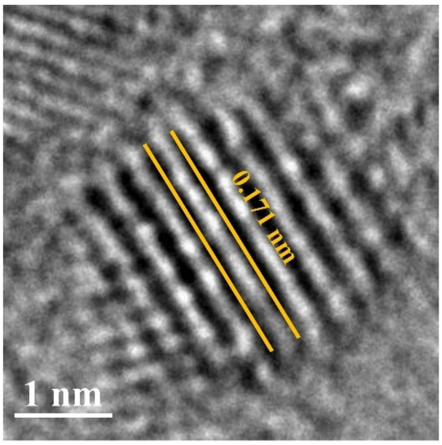

Fig. S3. The aberration corrected HAADF-STEM of Co-Ru@RuO_2 showing the Co phase.

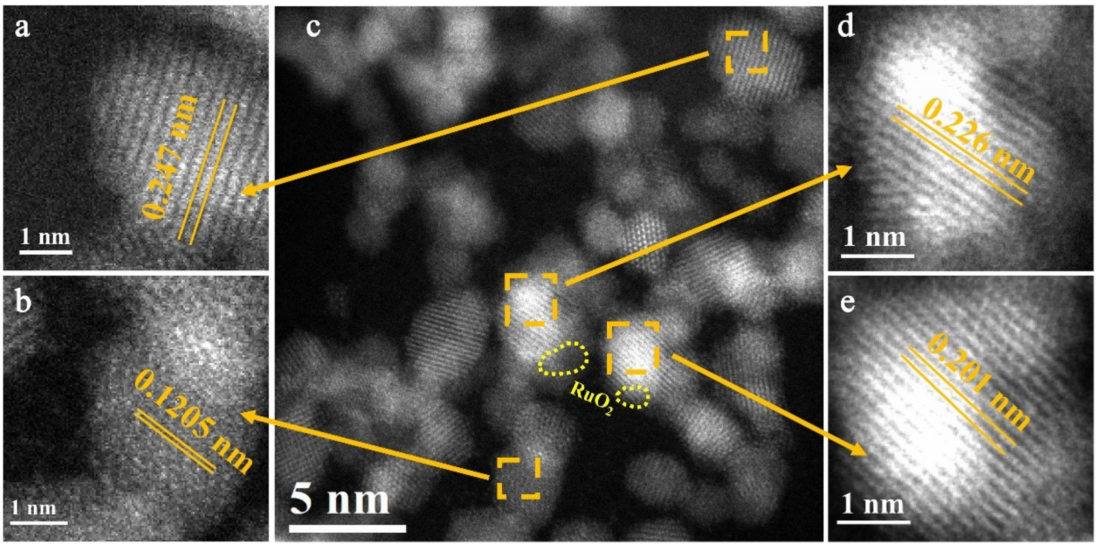

Fig. S4. The aberration-corrected HAADF-STEM images of Co-Ru@RuO_2.

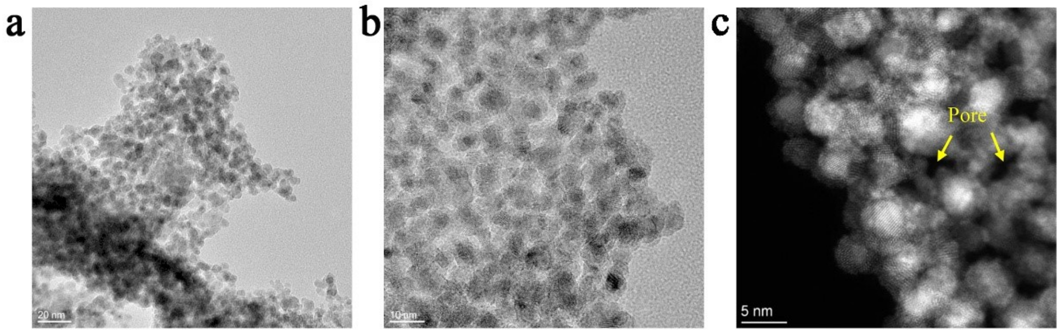

Fig. S5. a-c. High-magnification TEM and aberration-corrected HAADF-STEM of Co-Ru@RuO_2.

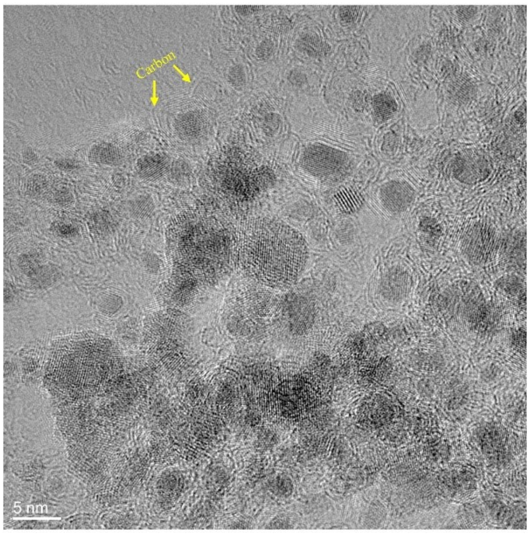

Fig. S6. The residual carbon in the Co-Ru@RuO_2.

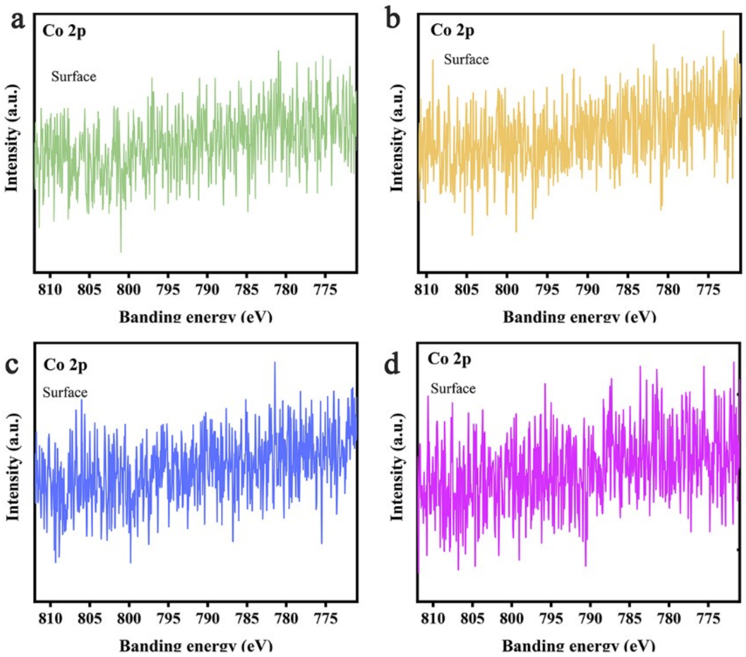

Fig. S7. a-d. XPS spectra of Co 2p in surface.

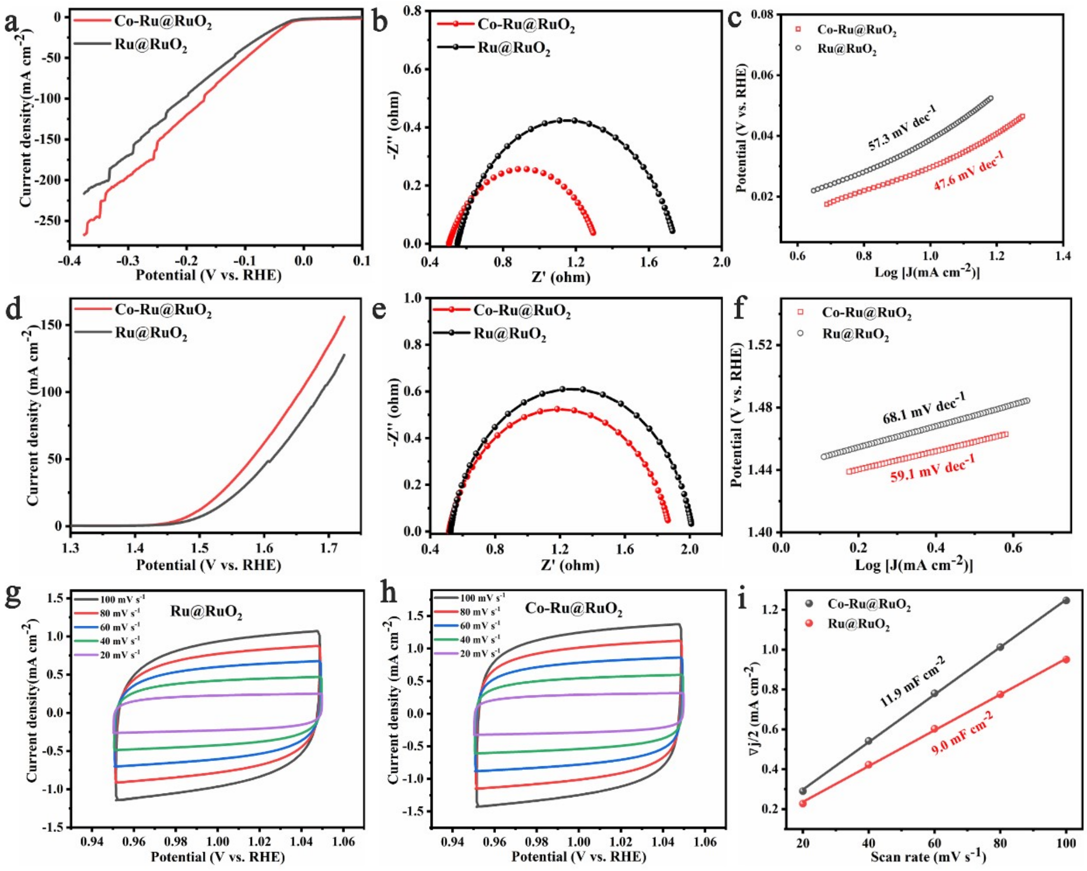

Fig. S8. The electrochemical studies of Ru@RuO_2 and Co-Ru@RuO_2 in 1.0 M KOH. **a.** HER polarization curves. **b.** EIS slopes of HER. **c.** Tafel slopes of HER. **d.** OER polarization curves. **e.** EIS slopes of OER. **f.** Tafel slopes of OER. **g.** The CV curves of Ru@RuO_2. **h.** The CV curves of Co-Ru@RuO_2. **i.** The C_{dl} values of Ru@RuO_2 and Co-Ru@RuO_2.

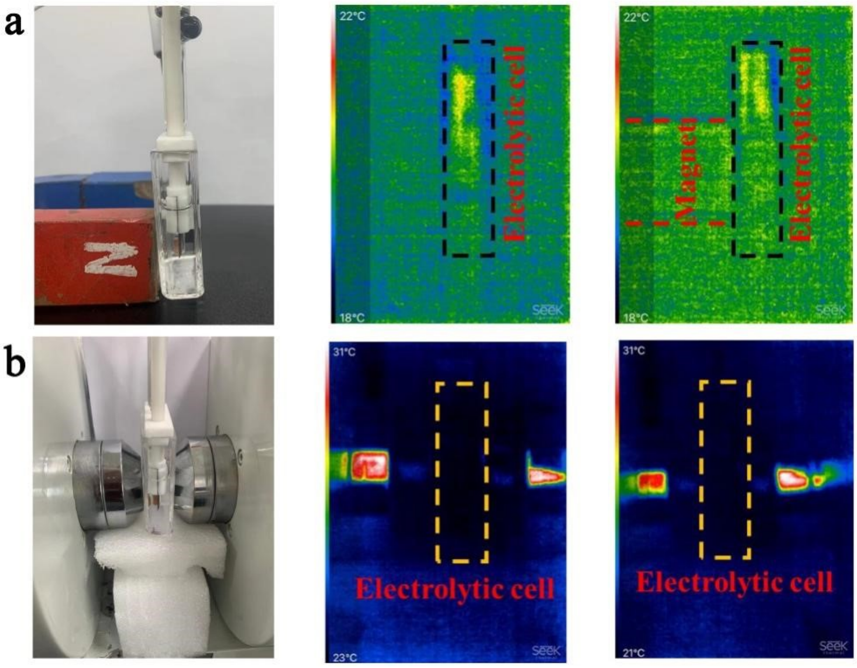

Fig. S9. **a-b.** The optical photos and infrared images of system under magnetic field.

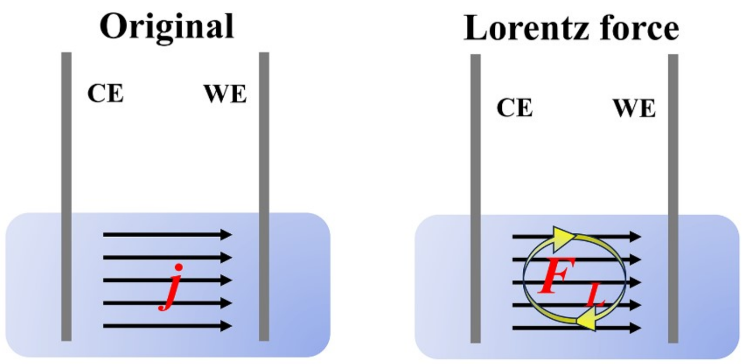

Fig. S10. Magnetohydrodynamics effects (Lorentz force) in a uniform external magnetic field.

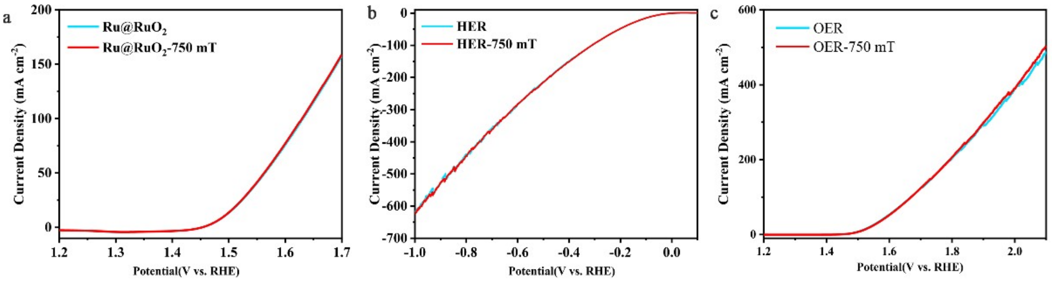

Fig. S11. a.Co-Ru@RuO_2 OER performance under an external magnetic field. b-c. HER and OER performance of Co-Ru@RuO_2 under an external magnetic field.

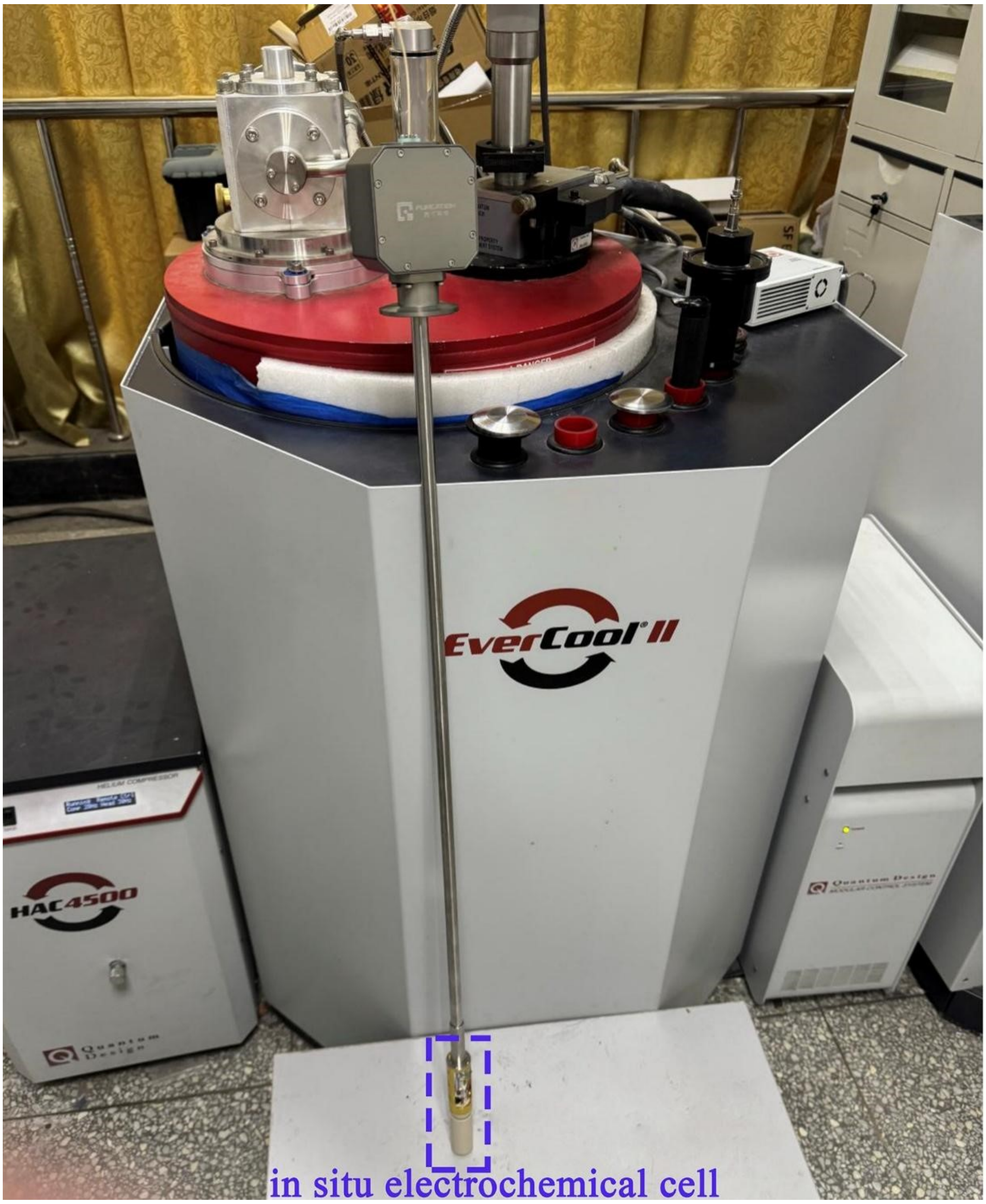

Fig. S12. The photo of PPMS in situ electrochemical cell.

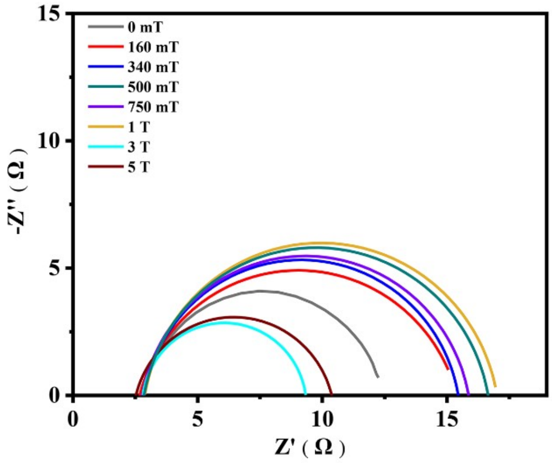

Fig. S13. EIS slopes in 1 M KOH under different magnetic field.

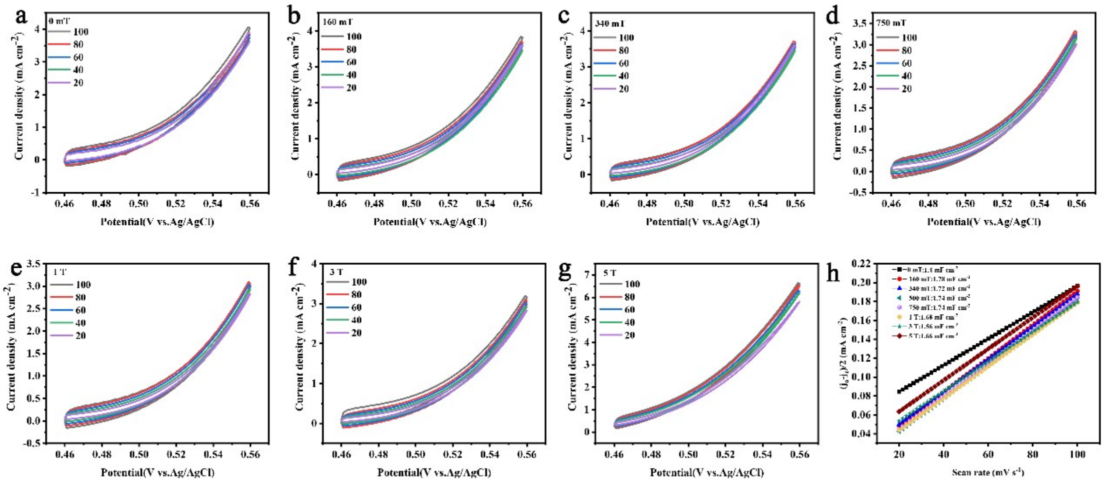

Fig. S14. a-g. The CV curves of Co-Ru@RuO2 under different magnetic fields. **h.** The C_{dl} values under different magnetic fields.

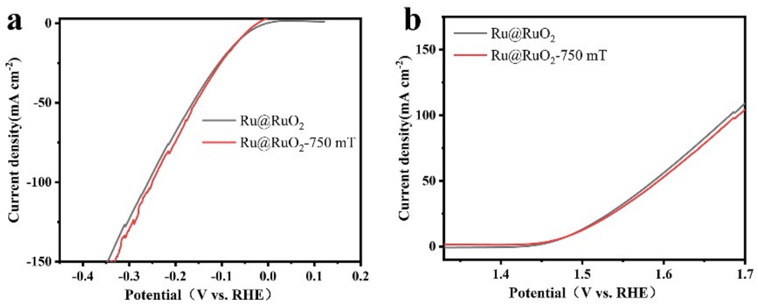

Fig. S15. The electrochemical studies of catalysts in 1.0 M KOH in 750 mT magnetic field**. a.** HER polarization curves. **b.** OER polarization curves.

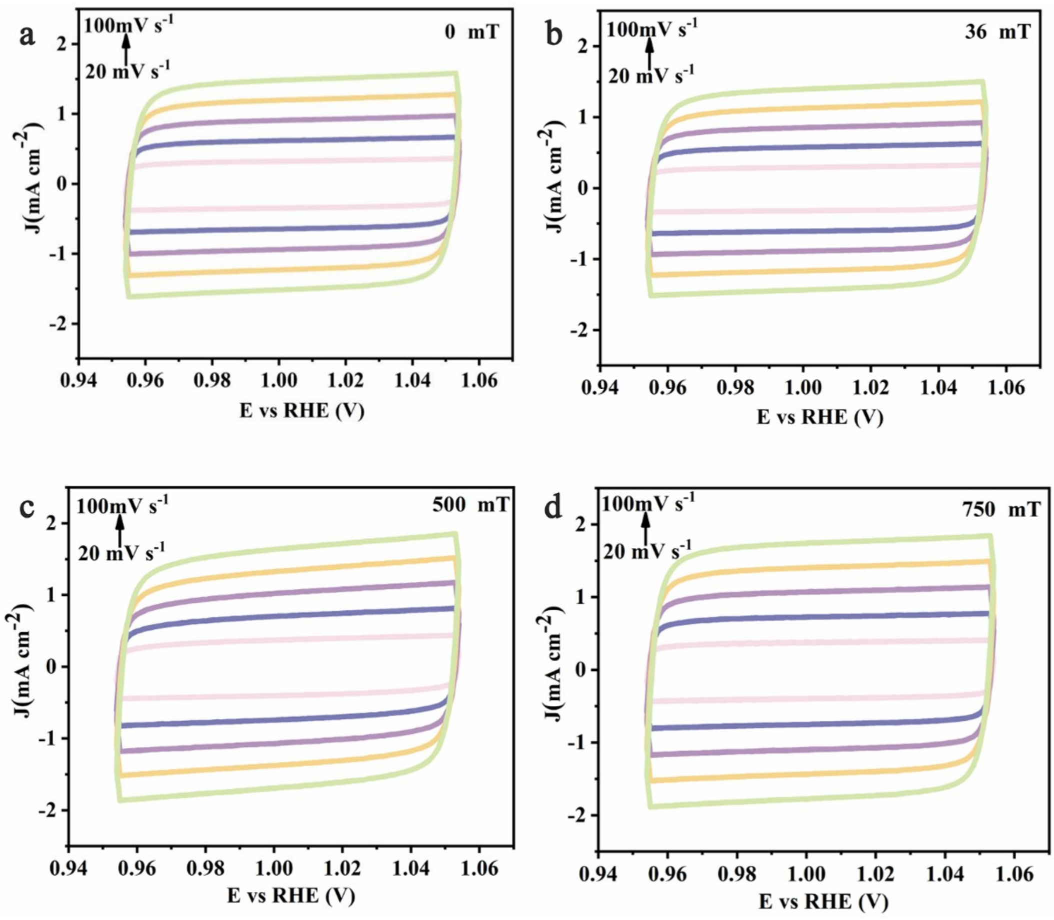

Fig. S16. The CV curves of Co-Ru@RuO_2 under different magnetic fields: **a**. 0 mT, **b** 36 mT, **c**. 500 mT, and **d**. 750 mT.

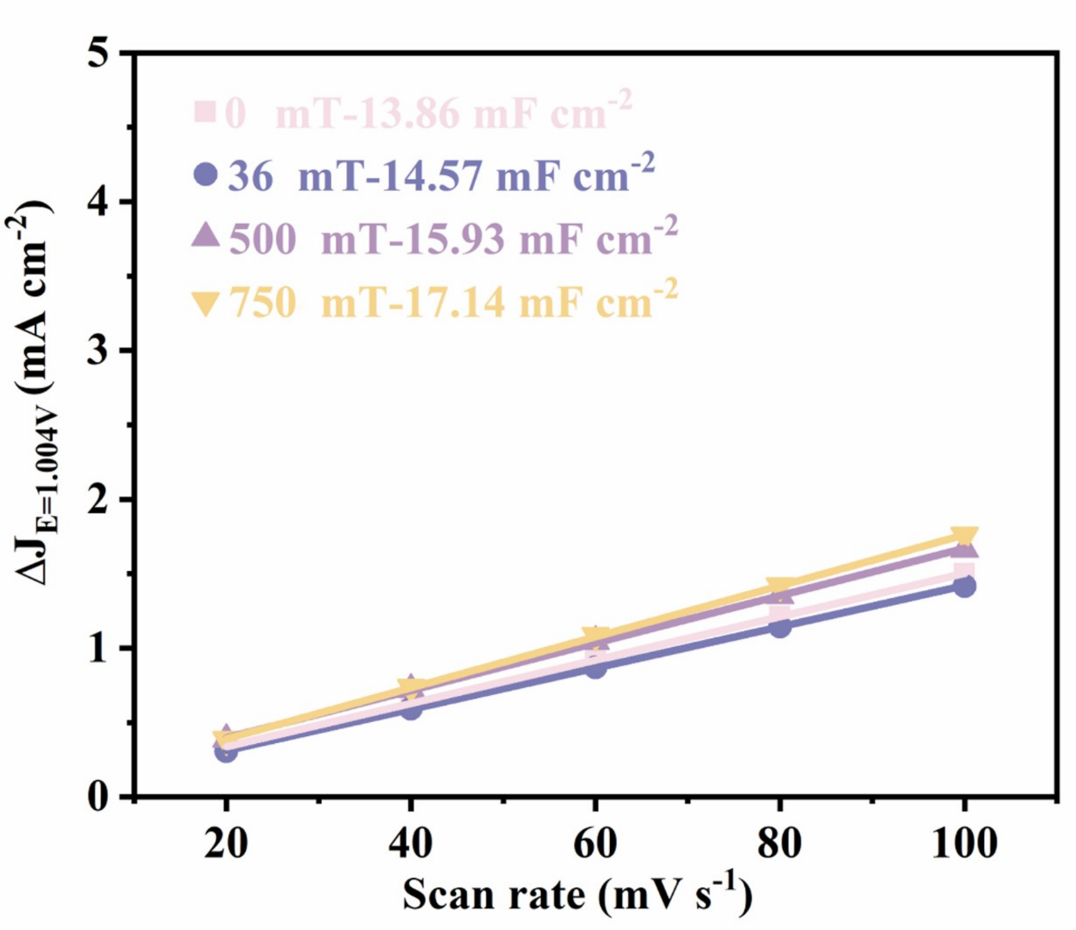

Fig. S17. The C_{dl} values under different magnetic fields.

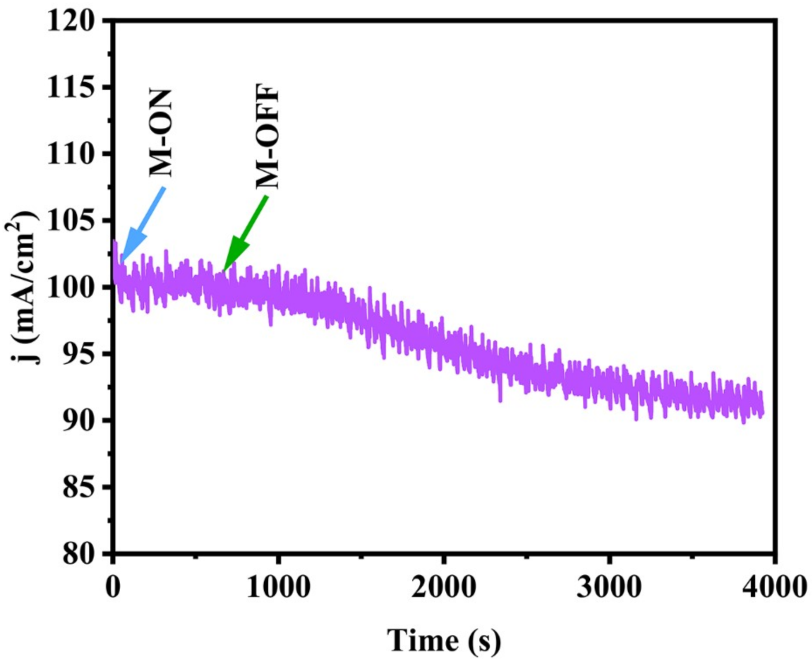

Fig. S18. The stability of the catalyst under a magnetic field.

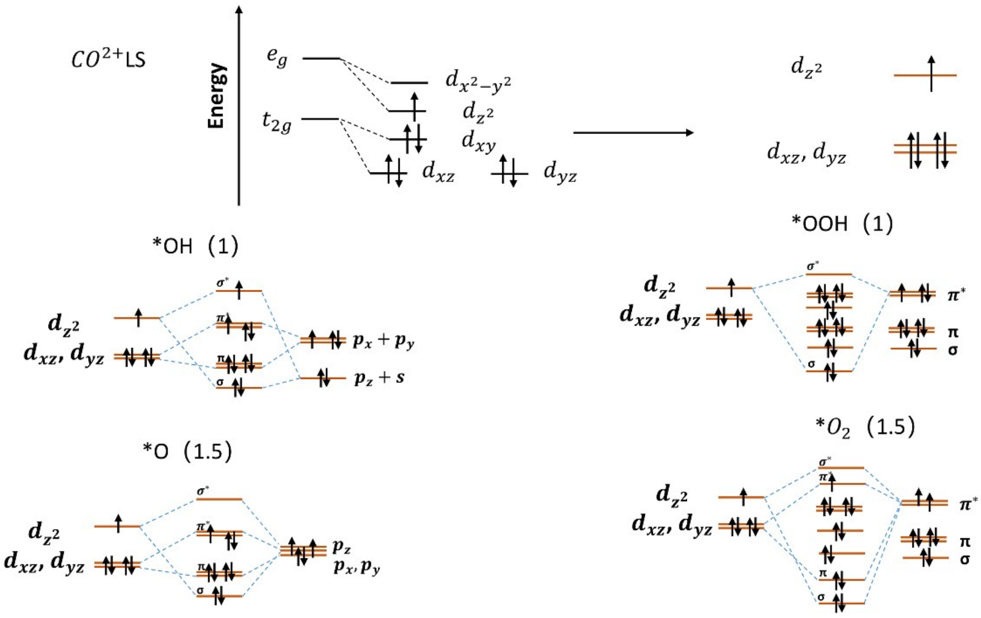

Fig. S19. The orbital interactions between low spin state of Co^{2+} cations and the OER intermediates.

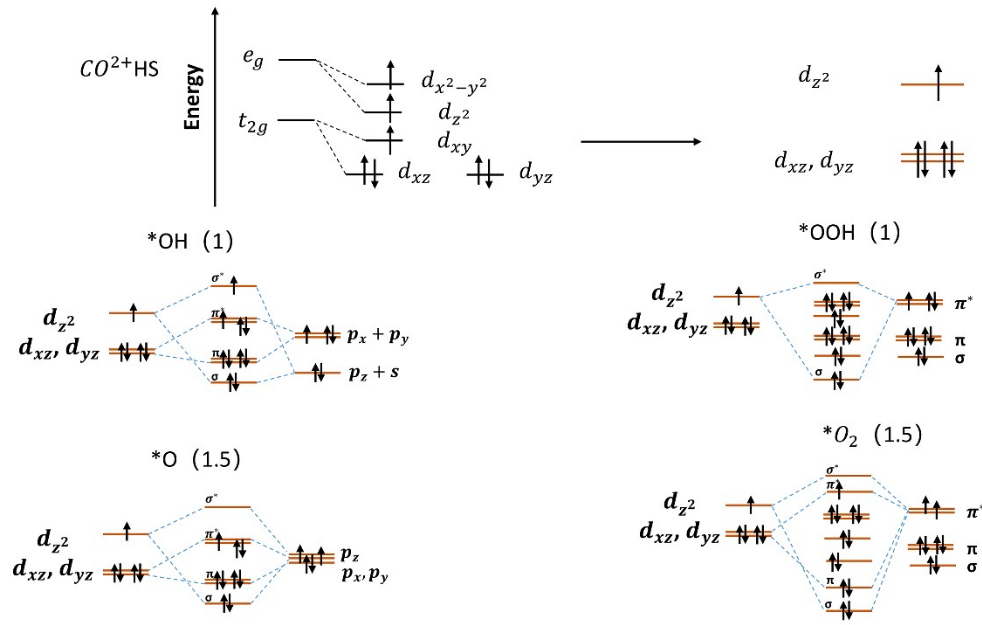

Fig. S20. The orbital interactions between high spin state of Co^{2+} cations and the OER intermediates.

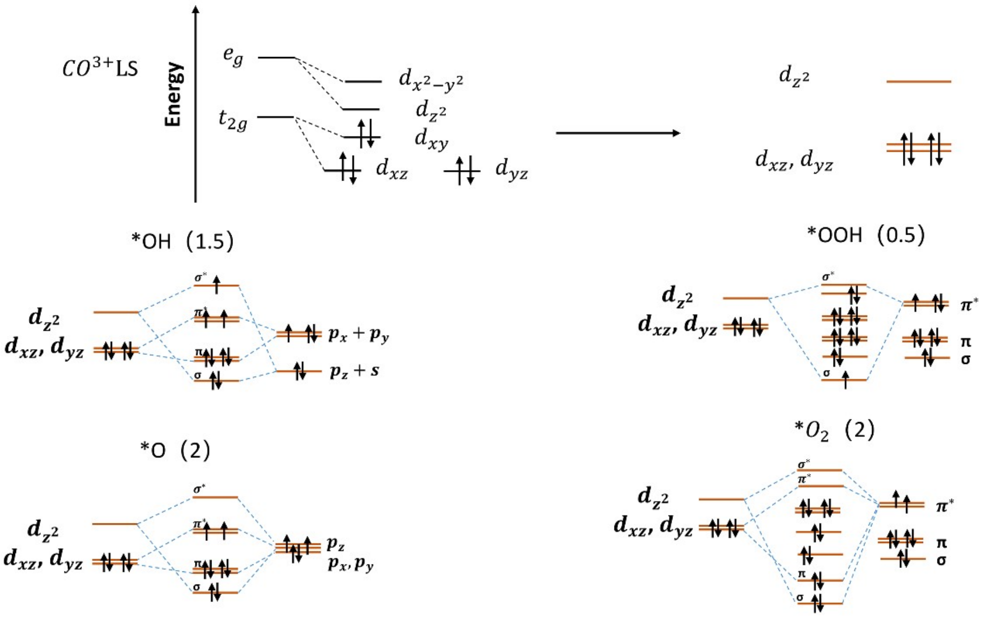

Fig. S21. The orbital interactions between low spin state of Co^{3+} cations and the OER intermediates.

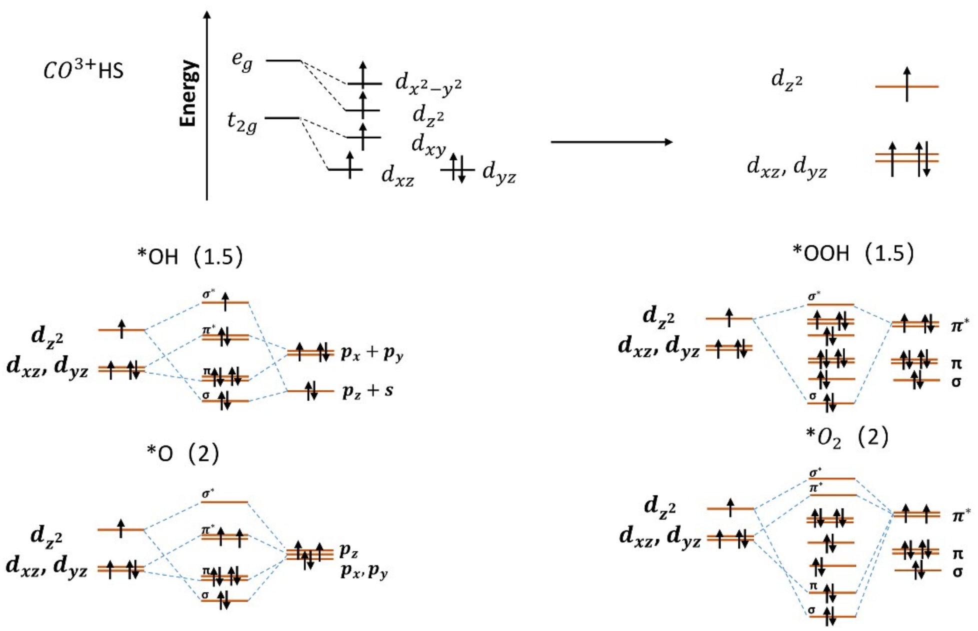

Fig. S22. The orbital interactions between high spin state of Co^{3+} cations and the OER intermediates.

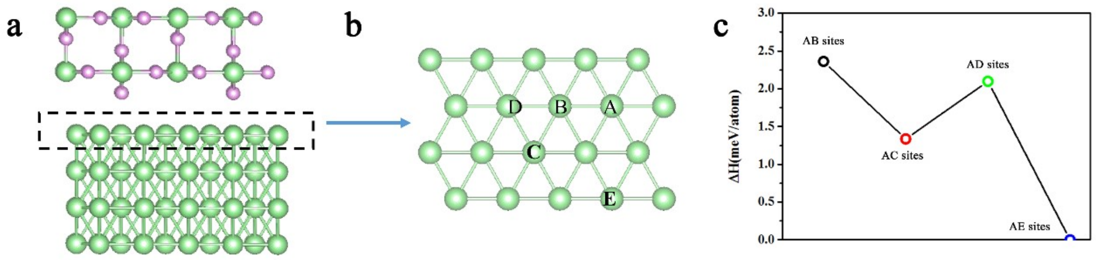

Fig. S23. **a**. The heterostructure model Ru(001)/RuO_2(110). **b**. Four possible doping models (AB sites, AC sites, AD sites, AE sites) for Ru(001)/RuO_2(110). **c**. Enthalpy difference (meV/atom) of four doping models.

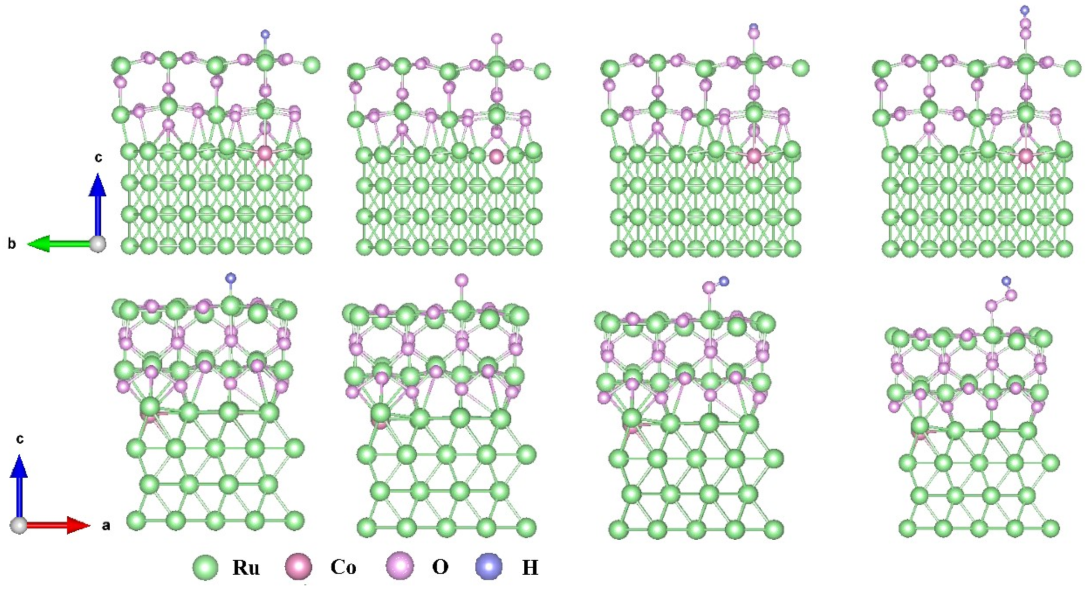

Fig. S24. The intermediates adsorption configurations for OER on the (110) surface of catalyst.

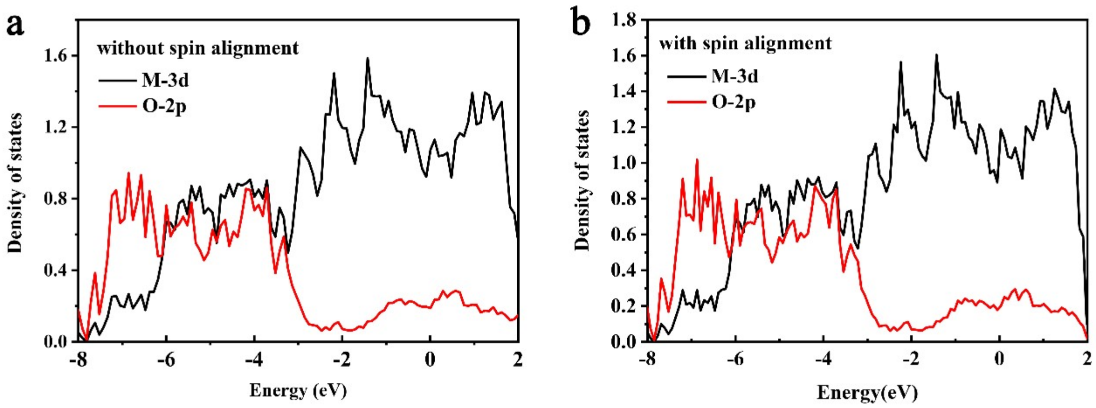

Fig. S25. The density of states (DOS) of catalyst without and with spin alignment.

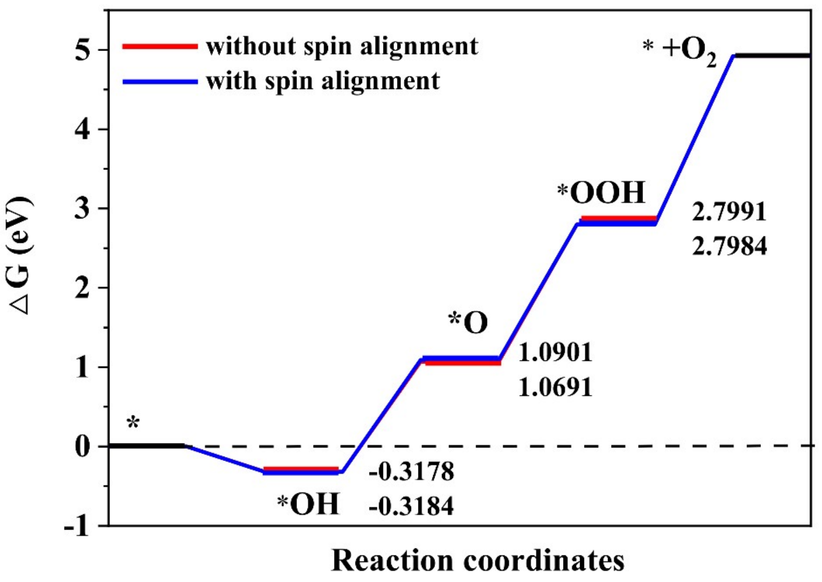

Fig. S26. The OER free energy diagram on catalyst with/without spin alignment.

Table S1. Comparison of the HER and OER performance of electrocatalysts based on external magnetic field reported in recent five years.

Electrocatalysts	Electrolyte	HER@10 $mA\cdot cm^{-2}$	OER@10 $mA\cdot cm^{-2}$	Magnetic field	Reference
Ni SMC	1M KOH	/	340 mV	300 mT	1
Cu–NiFe–LDHs	1M KOH	/	180 mV	800 mT	2
Ni/MoS_2	1M KOH	/	/	502 mT	3
Mn-RuO_2/NFs	0.5 M H_2SO_4	/	143 mV	330.9 mT	4
MoS_2	0.5 M H_2SO_4	113 mV	/	800 mT	5
$NiCo_2S_4$	1M KOH	104 mV	/	100 mT	6
1T-VSe_2	1M KOH	/	228 mV	800 mT	7
$CoSe_2$	1M KOH	/	239 mV	AC-4.1 mT	8
NiCoFe/MOF-74	1M KOH	/	214 mV	AC-5.184 mT	9
MoS_2	0.5 M H_2SO_4	105 mV	/	AC-3.25 mT	10
Pt_1/N-C	1M KOH	46 mV	/	/	11
Ru SAs–Ni_2P NPs	1M KOH	57 mV	/	/	12
Ru-MoO_2@PC/rGO	1M KOH	126 mV	/	/	13
Co3O4/CoFe2O4@NF	1M KOH	/	195.64 mV	100 mT	14
CFO@CoBDC/MCV	1M KOH	/	304 mV	600 mT	15
CoIr NCs	0.5M H2SO4	/	220 mV	106 mT	16
MCN	0.1M KOH	/	406 mV	350 mT	17
Mn-RuO2 NFs	0.5M H2SO4	/	143 mV	330.9 mT	18
NiFe/NiFeOOH core/shell NPs	1M KOH	/	209.2 mV	AC-3.9 mT	19
Ni3Fe-CW	1M KOH	76 mV	237 mV	300 mT	20
Pt NPs@C/Fe3O4/C	0.5M H2SO4	19.7 mV	/	AC-3.9 mT	21

Co-Ru@RuO_2	1M KOH	7 mV	250 mV	**750 mT**	**This work**
Co-Ru@RuO_2	1M KOH	/	260 mV	**3000 mT**	**This work**

Table S2. Spin configurations and spin quantum number of Co^{2+} and Co^{3+}.

Spin state	Oh Configuration	Spin quantum number
Co^{2+}(HS)	$(t_{2g})^5(e_g)^2$	S=3/2
Co^{2+}(LS)	$(t_{2g})^6(e_g)^1$	S=1/2
Co^{3+}(HS)	$(t_{2g})^4(e_g)^2$	S=2
Co^{3+}(LS)	$(t_{2g})^6(e_g)^0$	S=0

1. K. Saini, A. N. Nair, A. Yadav, L. G. Enriquez, C. J. Pollock, S. D. House, S. Yang, X. Guo and S. T. Sreenivasan, *Advanced Energy Materials*, 2023, **13**.
2. Z. Sun, L. Lin, J. He, D. Ding, T. Wang, J. Li, M. Li, Y. Liu, Y. Li, M. Yuan, B. Huang, H. Li and G. Sun, *J Am Chem Soc*, 2022, **144**, 8204-8213.
3. T. Sun, Z. Tang, W. Zang, Z. Li, J. Li, Z. Li, L. Cao, J. S. Dominic Rodriguez, C. O. M. Mariano, H. Xu, P. Lyu, X. Hai, H. Lin, X. Sheng, J. Shi, Y. Zheng, Y. R. Lu, Q. He, J. Chen, K. S. Novoselov, C. H. Chuang, S. Xi, X. Luo and J. Lu, *Nat Nanotechnol*, 2023, **18**, 763-771.
4. L. Li, J. Zhou, X. Wang, J. Gracia, M. Valvidares, J. Ke, M. Fang, C. Shen, J. M. Chen, Y. C. Chang, C. W. Pao, S. Y. Hsu, J. F. Lee, A. Ruotolo, Y. Chin, Z. Hu, X. Huang and Q. Shao, *Adv Mater*, 2023, **35**, e2302966.

5. W. D. Zhou, M. Y. Chen, M. M. Guo, A. J. Hong, T. Yu, X. F. Luo, C. L. Yuan, W. Lei and S. G. Wang, *NANO LETTERS*, 2020, **20**, 2923-2930.
6. X. B. Xu, X. M. Liu, W. Zhong, G. X. Liu, L. Zhang and Y. W. Du, *CERAMICS INTERNATIONAL*, 2023, **49**, 16836-16841.
7. M. Y. Chen, W. D. Zhou, K. Ye, C. L. Yuan, M. Y. Zhu, H. Yu, H. Z. Yang, H. Huang, Y. F. Wu, J. Y. Zhang, X. Q. Zheng, J. X. Shen, X. Wang and S. G. Wang, *SMALL*, 2023, **19**.
8. Y. Ding, W. D. Zhou, X. F. Luo, J. L. Huang, D. Q. Peng, M. Y. Chen, H. Zhou, C. Hu and C. L. Yuan, *APPLIED PHYSICS LETTERS*, 2022, **121**.
9. H. B. Zheng, Y. L. Wang, P. Zhang, F. Ma, P. Z. Gao, W. M. Guo, H. Qin, X. P. Liu and H. N. Xiao, *CHEMICAL ENGINEERING JOURNAL*, 2021, **426**.
10. M. X. Su, W. D. Zhou, L. Liu, M. Y. Chen, Z. Z. Jiang, X. F. Luo, Y. Yang, T. Yu, W. Lei and C. L. Yuan, *ADVANCED FUNCTIONAL MATERIALS*, 2022, **32**.
11. S. Fang, X. Zhu, X. Liu, J. Gu, W. Liu, D. Wang, W. Zhang, Y. Lin, J. Lu, S. Wei, Y. Li and T. Yao, *Nat Commun*, 2020, **11**, 1029.
12. K. Wu, K. Sun, S. Liu, W.-C. Cheong, Z. Chen, C. Zhang, Y. Pan, Y. Cheng, Z. Zhuang, X. Wei, Y. Wang, L. Zheng, Q. Zhang, D. Wang, Q. Peng, C. Chen and Y. Li, *Nano Energy*, 2021, **80**, 105467.
13. J. Y. Han, S. H. Cai, J. Y. Zhu, S. Yang and J. S. Li, *Chem Commun (Camb)*, 2021, **58**, 100-103.
14. Y. Ma, Y. Zhou, C. Wang, B. Gao, J. Li, M. Zhu, H. Wu, C. Zhang and Y. Qin, *Advanced Materials*, 2023, **35**.
15. S. Ma, K. Wang, M. Rafique, J. Han, Q. Fu, S. Jiang, X. Wang, T. Yao, P. Xu and B. Song, *Angewandte Chemie International Edition*, 2024, **63**.
16. L. Li, Y. Wang, R. R. Nazmutdinov, R. R. Zairov, Q. Shao and J. Lu, *Nano Letters*, 2024, **24**, 6148-6157.
17. J. Yan, Y. Wang, Y. Zhang, S. Xia, J. Yu and B. Ding, *Advanced Materials*, 2020, **33**.
18. L. Li, J. Zhou, X. Wang, J. Gracia, M. Valvidares, J. Ke, M. Fang, C. Shen, J. M.

Chen, Y. C. Chang, C. W. Pao, S. Y. Hsu, J. F. Lee, A. Ruotolo, Y. Chin, Z. Hu, X. Huang and Q. Shao, *Advanced Materials*, 2023, **35**.

19. D. Peng, C. Hu, X. Luo, J. Huang, Y. Ding, W. Zhou, H. Zhou, Y. Yang, T. Yu, W. Lei and C. Yuan, *Small*, 2022, **19**.

20. Y. Wang, Y. Shang, Z. Cao, K. Zeng, Y. Xie, J. Li, Y. Yao and W. Gan, *Chemical Engineering Journal*, 2022, **439**.

21. H. Zhan, Z. Jiang, X. Luo, Y. Huang, D. Ye, W. Xu, C. Hu, H. Zhou, W. Lei and C. Yuan, *Advanced Functional Materials*, 2024, **34**.
